# Characteristic radiological findings indicating the possible involvement of the hepatic hilar lymph nodes in patients with colorectal liver metastasis: Case report

**DOI:** 10.1016/j.ijscr.2020.05.040

**Published:** 2020-05-29

**Authors:** Yota Kawasaki, Satoshi Iino, Tetsuya Idichi, Shinichi Ueno, Shoji Natsugoe

**Affiliations:** Department of Digestive Surgery, Breast and Thyroid Surgery, Graduate School of Medical Sciences, Kagoshima University, Japan

**Keywords:** CRCLM, colorectal cancer liver metastasis, LNs, lymph nodes, S, segment, CT, computed tomography, PET, positron emission tomography, DWI, diffusion-weighted imaging, MRI, magnetic resonance imaging, SUV, standardized uptake value, Colorectal cancer liver metastasis, Hepatic hilar lymph node involvement

## Abstract

**Background:**

Colorectal cancer liver metastasis (CRCLM) concomitant with infiltration of lymph nodes (LNs) in the hepatic pedicle is difficult to manage, and is regarded as an extrahepatic metastasis; undertaking hepatectomy is controversial in such a scenario.

**Case presentation:**

Case 1, A 55-year-old woman was diagnosed with multiple liver metastases arising from rectal cancer along with enlargement of a retropancreatic LN. A characteristic image prior to hepatectomy demonstrated evident tumor progression from a metastatic lesion in segment 5 of the liver (S5) to the hepatic hilum along with Glisson 5. Post-operative histopathological examination revealed viable adenocarcinoma cancer cells originating from rectal cancer in all the liver metastatic lesions and retropancreatic LN.

Case 2, A 89-year-old woman was diagnosed with transverse colon cancer with multiple liver metastases. Radiological examination before hepatectomy revealed tumor progression from a metastatic lesion in the segment 6 of the liver (S6) to the hepatic hilum along with Glisson 6 and LN involvement in the hepatoduodenal ligament. Post-operative histopathological examination demonstrated adenocarcinoma cancer cells in the liver metastatic lesions and in a hepatoduodenal LN.

**Conclusion:**

When encountering tumor progression from liver metastatic lesions to hepatic hilum along with its Glisson branch, the possibility of hepatic hilar LN involvement should be considered. Indeed, the surgical management of CRCLM with hepatic hilar LN involvement is controversial, but could be acceptable if the positive LNs are limited to the hepatic pedicle and retropancreatic area.

## Highlights

• Detection of colorectal cancer liver metastasis (CRCLM) with the infiltration of lymph nodes (LNs) in the hepatic pedicle is not so simple.
• When detecting tumor progression from CRCLM along with Glisson branch, the possibility of hepatic hilar LNs involvement should be considered.
• Surgical approach against CRCLM with hepatic hilar LNs involvement is controversial, but could be acceptable.

## 1 Introduction

Colorectal cancer liver metastasis (CRCLM) accompanied by metastatic infiltration of LNs in the hepatic pedicle is regarded as extrahepatic metastasis, and hepatectomy is considered controversial in this case. Indeed, it is frequently considered as a contraindication for hepatectomy because of the poor prognosis [1]. However, several reports also support hepatectomy and lymphadenectomy to improve survival rates [2]. Therefore, careful consideration is required for deciding whether surgery is indicated in such a complex situation.

We report two cases of CRCLM with hepatic hilar LN involvement. Both the cases had characteristic radiological findings indicating the possible involvement of the hepatic hilar LNs. We would like to especially focus on the characteristic preoperative images and the mechanism of metastasis from metastatic liver lesions to hepatic hilar LNs.

This work has been reported in line with the SCARE criteria (Agha) [3].

## 2 Case presentations

### 2.1 Case 1

**Fig. 1:**
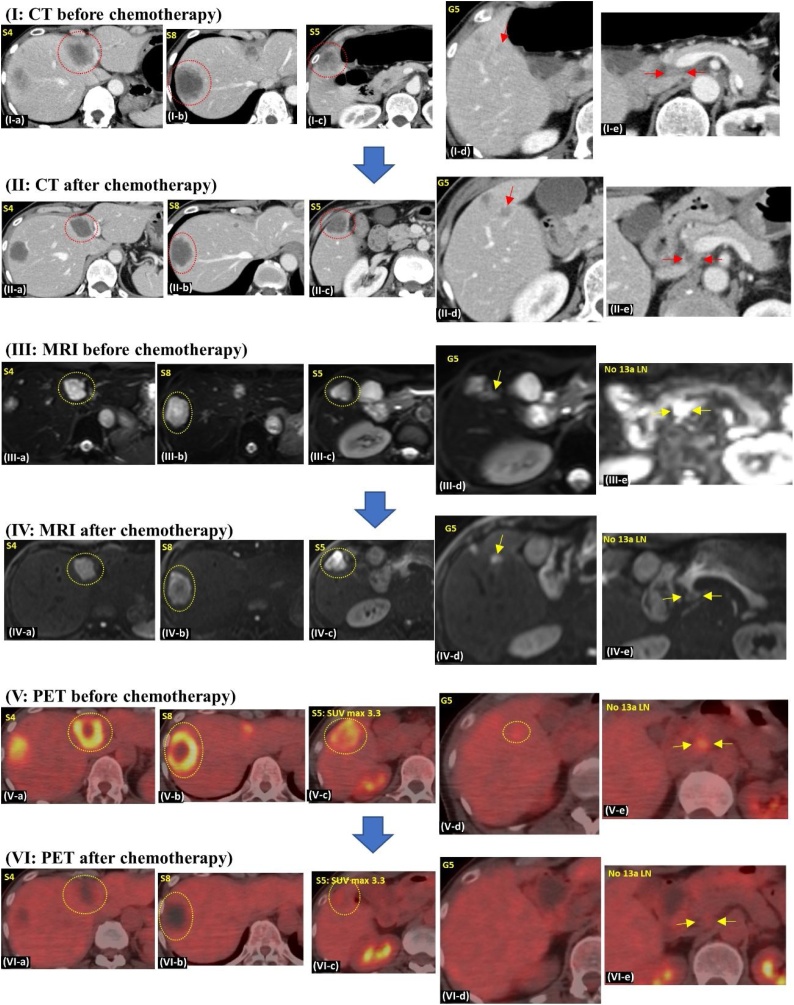
I-a, b, c) Three metastatic lesions noted in the liver (S4: size 44 mm, S8: size 45 mm, and S5: size 35 mm). I-d) Suspected tumor progression along Glisson 5 (red arrow). I-e) A retropancreatic LN of size 12 mm is seen (red arrow). II-a, b, c) All liver metastatic lesions reduced in size (S4: size 30 mm, S8: size 40 mm, S5: size 28 mm). II-d) Tumor progression along Glisson 5 is more evident (red arrow). II-e) Retropancreatic LN have reduced in size (10 mm; red arrow). III-a, b, c) DWI-MRI revealing high intensity areas concomitant with the liver metastases. III-d) DWI-MRI showing high intensity signal along Glisson 5. III-e) DWI of MRI showing high intensity signals in the retropancreatic LN. IV-a, b, c, d, e) DWI-MRI revealing high intensity signals consistent with suspected metastatic lesions. V-a, b, c, d, e) PET revealing high intensity signals, all consistent with suspected metastatic lesions including retro-pancreatic LN. VI-a, b, c) Although S5 metastasis still shows a weak intensity signal, there is no detectable signal intensity in the S4 and S8 metastases. VI-d, e) PET does not show any signal intensity in Glisson 5 and the retropancreatic LN.

**Fig. 2:**
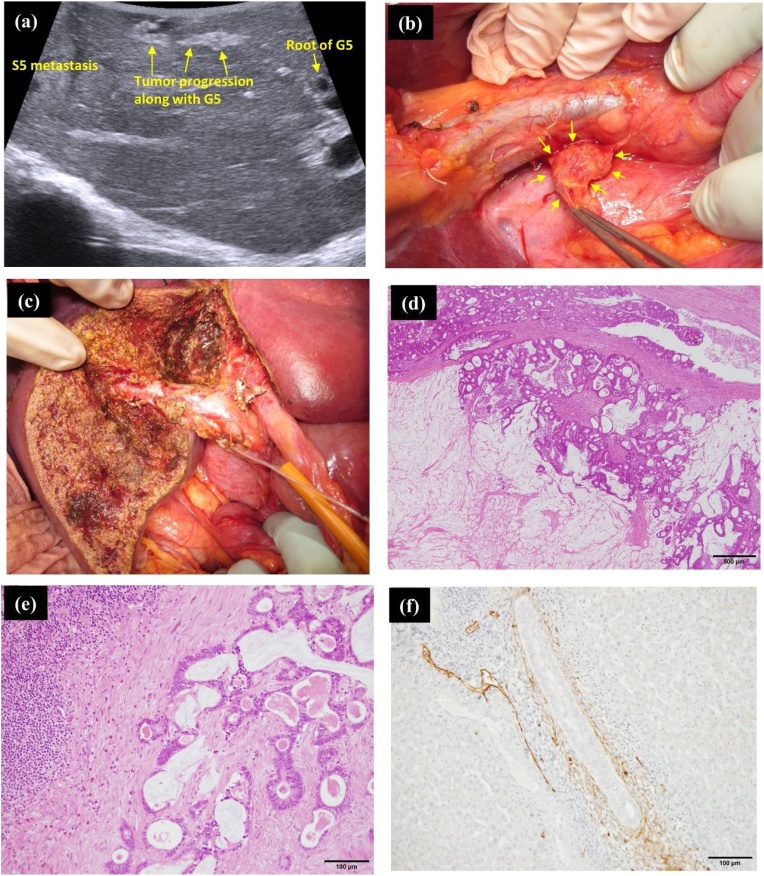
(a) Intraoperative ultrasound clearly showing suspected tumor progression along Glisson 5. However, it has not reached the root of Glisson 5. (b) An enlarged retroperitoneal LN is observed. (c) Surgical field after anatomical liver S4 and S5 sub-segmentectomy. (d) On histopathological examination, viable adenocarcinoma cells are detected in all the liver metastatic lesions. (e) Viable adenocarcinoma cells are detected in the resected retropancreatic LN. (f) Immunohistological staining using D2-40 antibody showing cancer cells in the lymphatic duct of Glisson 5.

A 55-year-old woman was referred to our institution with multiple liver metastases from rectal cancer along with the enlargement of a retropancreatic lymph node. She was treated with laparoscopic high anterior resection of the rectum, followed by chemotherapy in the previous hospital. Pathological examination revealed a stage IVA (pT4a, pN2a, pM1a) rectal cancer based on the 8th edition of the Union for International Cancer Control (UICC). On comparison of the pre- and post-chemotherapeutic computed tomography (CT) images, the size of the liver metastasis in segment 8 of the liver (S8) had reduced from 45 mm to 40 mm, metastasis in S4 reduced from 44 mm to 30 mm, and metastasis in S5 reduced from 35 mm to 28 mm following chemotherapy (Fig. 1; I-a∼c and II-a∼c). Additionally, the retropancreatic lymph node reduced in size from 12 mm to 10 mm (Fig. 1; I-e and II-e). Regarding the positron emission tomography (PET) evaluation, the signal intensity was strong in all the liver metastases and retropancreatic LN prior to chemotherapy (Fig. 1; V-a∼e). However, there was an absence of signal intensity in the liver metastatic lesions with the exception of the S5 metastasis after chemotherapy (Fig. 1; VI-a∼e). On the diffusion-weighted imaging (DWI) of magnetic resonance imaging (MRI), apart from the liver metastases and retropancreatic LN, Glisson 5 also displayed signal hyperintensity both, before and after chemotherapy (Fig. 1; III-a∼e and IV-a∼e). This was suspected to be tumor progression from the S5 liver metastasis. Based on the above evaluation, we decided to perform anatomical sub-segmentectomy of S4 and S5, partial resection of S8, and sampling of the enlarged retropancreatic LN (Fig. 2c). As a notable intraoperative finding, we could see an enlarged and echogenic Glisson 5, which probably came from the adjoining S5 metastasis, but did not reach the root of Glisson 5 (Fig. 2a). In addition, we could see enlarged retropancreatic LN as expected, and performed sampling from it (Fig. 2b). On histopathological examination, both, viable and necrotic adenocarcinoma cancer cells from rectal cancer were detected in the metastatic liver lesions, Glisson 5, and the retro-pancreatic LN (Fig. 2e). Additional immunohistological evaluation using D2-40 antibody demonstrated cancer cells within the lymphatic duct of Glisson 5 (Fig. 2f). During the post-operative period, the patient received adjuvant chemotherapy for six months. Although no recurrence was evident during adjuvant chemotherapy, multiple lung metastases were detected after that period, prompting further administration of intensive chemotherapy, which has controlled the metastatic lesions to date. Despite having advanced rectal cancer, she has been able to survive for more than 20 months since the diagnosis owing to multidisciplinary treatment.

### 2.2 Case 2

**Fig. 3:**
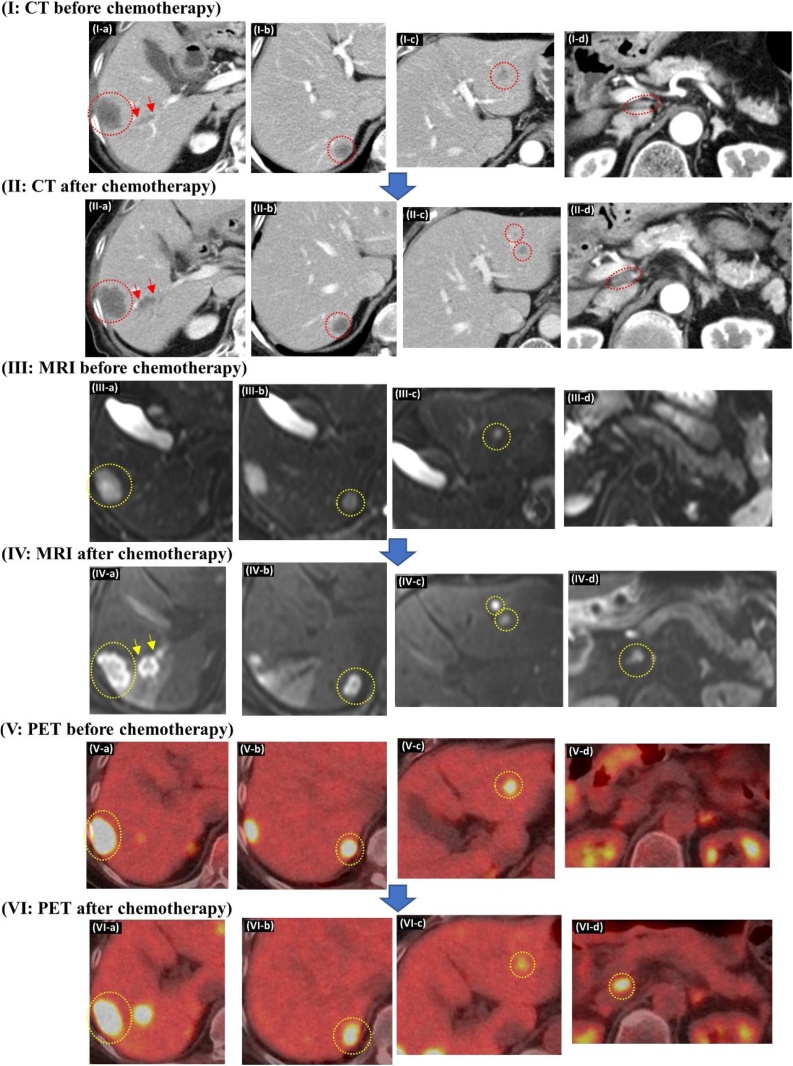
I-a, b, c) Three liver metastatic lesions (S6: size 35 mm, S7: size 23 mm, S3: size 13 mm). In addition, suspected tumor progression is seen along Glisson 6 (red arrow). I-d) Small LN detected in the hepatoduodenal ligament. II-a, b, c) In addition to the previous three liver metastatic lesions, one more metastasis is detected in S3. Furthermore, suspected tumor progression along Glisson 6 has become more evident (red arrow). II-d) LN in the hepatoduodenal ligament have increased in size when compared to the CT before chemotherapy. III-a, b, c, d) DWI-MRI displayed high intensity signals consistent with liver metastatic lesions. However, no signal intensity has been detected in the hepatoduodenal LN. IV-a, b, c) DWI-MRI shows high intensity in not only all the liver metastatic lesions including the new S3 metastasis, but also in Glisson 6 and the hepatoduodenal LN (yellow arrow). V-a, b, c, d) PET revealing high intensity signal consistent with all the liver metastatic lesions and Glisson 6, but not in the hepatoduodenal LN. VI-a, b, c, d) PET shows high intensity signals not only in all the liver metastatic lesions, but also Glisson 6 and the hepatoduodenal LN.

**Fig. 4:**
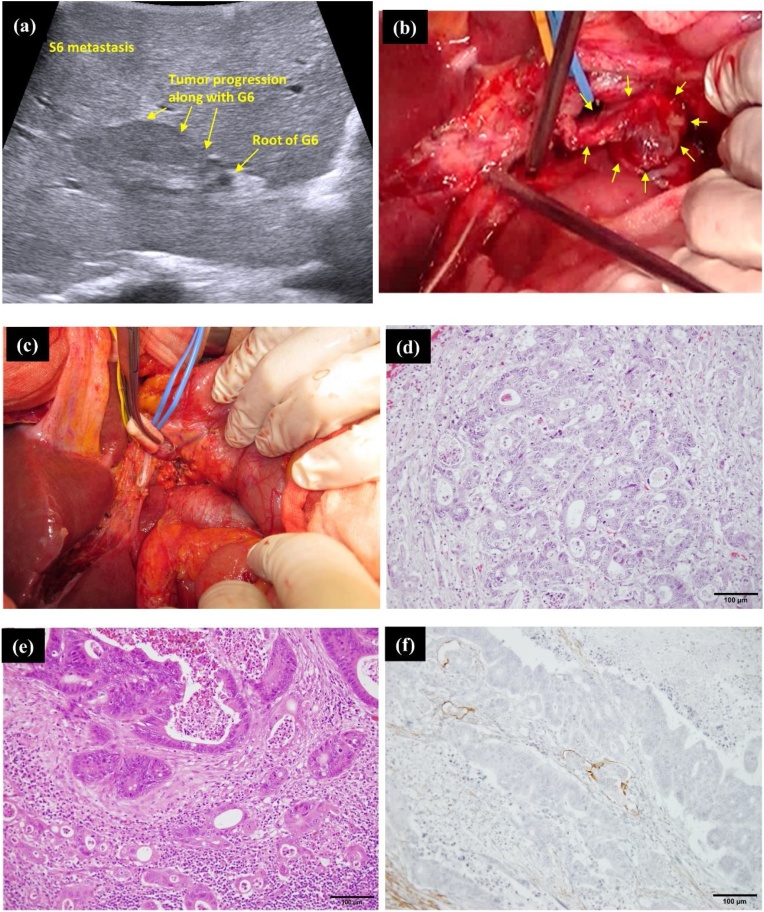
(a) Intraoperative ultrasound clearly showing suspected tumor progression along Glisson 6. However, it has not reached the root of Glisson 6. (b) An enlarged LN is observed at the hepatoduodenal ligament (yellow arrow). (c) Surgical field after dissection of the hepatoduodenal area. (d) On histopathological examination, viable adenocarcinoma cells are seen in all the liver metastatic lesions. (e) Viable adenocarcinoma cells detected in the resected hepatoduodenal LN. (f) Immunohistological staining using D2-40 antibody shows cancer cells in the lymphatic duct of Glisson 6.

A 89-year-old woman diagnosed as having transverse colon cancer with regional LN metastases and multiple liver metastases (total 2 liver metastases; S6: size 30 mm, S7: size 13 mm) was transferred to our institution. Therefore, we decided to perform colorectomy first, followed by hepatectomy as a secondary surgery considering the patient’s poor physical status. Pathological examination after laparoscopic transverse colorectomy with regional LN dissection indicated a stage IVA (pT3[SS], pN1a, pM1a [HEP]) transverse colon adenocarcinoma based on the 8th edition of the UICC. Subsequent to colorectomy, radiographic images for re-evaluation revealed additional liver metastatic lesions in S3 in addition to the pre-existing ones in S6 and S7 (total 3 liver metastases; S6: size 35 mm, S7: size 23 mm, S3: size 13 mm), along with suspected tumor progression along Glisson 6, which probably came from the adjoining S6 metastasis (Fig. 3; I-a∼c). Consequently, additional chemotherapy was administered prior to the impending hepatectomy. However, post-chemotherapeutic examination by CT and MRI revealed an additional S3 liver metastasis (total 4 liver metastases; S6: size 35 mm, S7: size 17 mm, S3: size 9 mm and 7 mm) (Fig. 3; II-a∼c and III-a∼c). Positron emission tomography-computed tomography (PET-CT) detected standardized uptake value (SUV) accumulation in a hepatoduodenal LN, considered as tumor metastasis for the first time (Fig. 3; VI-d). The tumor progression along Glisson 6 also became more evident on comparing images from before and after chemotherapy (Fig. 3; I∼VI-a). There was a re-evaluation of the treatment plan, to decide between surgery or continuous chemotherapy. Eventually, the decision was made to perform hepatectomy with LN dissection of hepatoduodenal area. Soon after discussion, liver posterior segmentectomy, partial resection of S3, and LN dissection of hepatoduodenal area was performed. Histopathological examination revealed adenocarcinoma cancer cells from colon cancer, that were detected not only in all the liver metastatic lesions detected by preoperative imaging, but also in the hepatoduodenal LN (Fig. 4d). In addition, metastatic cancer cells from liver segment S6 invaded and progressed along Glisson 6, consistent with the preoperative images (Fig. 4e). Additional immunohistological evaluation using D2-40 antibody demonstrated cancer cells within the lymphatic duct of Glisson 6 (Fig. 4f). During the post-operative period, the patient could not receive adjuvant chemotherapy owing to severe fatigue. Therefore, a single metastatic lesion was detected in the lung, with two remnant metastatic lesions in the liver at 4 months after hepatectomy. Intensive chemotherapy was administered; it has controlled the metastases to date. Despite having advanced cancer of the transverse colon, she has survived for more than 16 months since the diagnosis owing to multidisciplinary treatment.

## 3 Discussion

We report two cases of CRCLM with hepatic hilar LN involvement; these have helped us gain useful insight into the treatment of CRCLM patients.

Firstly, detection of positive LN involvement around the hepatic hilum itself is not so simple. Initially, hepatic hilar LN involvement by means of CT and MRI scans was not evident, because of the small size. Even if LNs around the hepatic hilum could be detected, it is very difficult to distinguish tumor positive or negative LNs, because it is not unusual to notice enlarged LNs in this area. Only after conducting a PET evaluation, we could detect hepatic hilar LN involvement. Certainly, PET is supposed to be one of the most powerful tools for detection of cancer metastasis, but it is available only in certain specific institutions such as high volume hospitals. Therefore, it is important to find radiological evidence of possible LN metastases around the hepatic hilar area with a CT or MRI in cases of CRCLM.

Secondly, the issue of whether the hepatic hilar LN involvement arises from the primary colorectal tumor lesion or the liver metastatic site as second-degree metastases, remains unclear. It is probable that the metastases to the hepatic hilar LNs originated from the liver metastases and not from the primary colorectal tumor lesion in the above two cases [4,5]. As shown in Fig. 1, Fig. 3, immunohistological evaluation of the Glisson branch using D2-40 antibody detected cancer cells in its lymphatic duct. Considering this fact, the following mechanism may be proposed to be acceptable. In the beginning, metastatic cancer cells in the liver invaded and progressed along the intrahepatic Glisson sheath. Subsequently, the cancer cells progressed and spread from the lymphatic duct of the Glisson sheath to the lymphatic duct of the hepatoduodenal ligament. Finally, cancer cells reached the hepatic hilum and the hepatoduodenal LNs and established metastasis. When we carefully examined the preoperative images once again keeping this theory in mind, both cases showed tumor progression along the Glisson branch. DWI images of MRI clearly showed high intensity in the Glisson branch due to suspected tumor metastasis progression. Accordingly, tumor progression along the Glisson branch and hepatic hilar LN involvement may have a close relationship. Therefore, if we encounter tumor progression along the intrahepatic Glisson branch, we may have to consider the possibility of hepatic hilar LN involvement, even if an enlarged lymph node is not evident.

Thirdly, hepatectomy with lymphadenectomy in patients with CRCLM together with hepatic hilar LN involvement should be considered as one potential therapeutic approach, especially if positive LNs are limited to the area around the hepatoduodenal ligament and the retropancreatic area. Jaeck et al. and Adam et al. emphasized that the location of the positive LNs significantly affected the survival rate [1,6]. According to their reports, patients with positive LNs in the hepatic pedicle and retropancreatic area had significantly better prognosis than patients with positive LNs around the common hepatic artery and celiac axis. Despite the above evidence, the type of lymphadenectomy, whether systemic or sampling, is controversial. However, surgeons should not abandon surgical approach even in case of CRCLM with hepatic hilar LN involvement [7].

## 4 Conclusion

When tumor progression is encountered from liver metastatic lesions to the hepatic hilum along its Glisson branch, the possibility of hepatic hilar LN involvement has to be considered. Indeed, surgical management of CRCLM with hepatic hilar LN involvement is controversial, but could be acceptable if the positive LNs are limited to the hepatic pedicle and retropancreatic area.

## Declaration of Competing Interest

None of the authors have any commercial or financial involvement in connection with this study that represents or appears to represent any conflicts of interest.

## Sources of funding

None.

## Ethical approval

Case reports are exempted from ethical approval according to our institution policies.

## Consent

Written informed consent was obtained from both of the patients for publication of this Case report and any accompanying images.

## Author contribution

Kawasaki Y, Satoshi I and Idichi T: Conception of reporting case, data recording, and drafting.

Kawasaki Y, Satoshi I and Idichi T: Management of case.

Kawasaki Y, Sakoda M, Tanonue K, Kurahara H, Mataki Y, Maemura K, Ueno S and Natsugoe S: Critical revision of article and final approval of the version to be submitted.

## Registration of research studies

Not applicable.

## Guarantor

Kawasaki Y.

## Provenance and peer review

Not commissioned, externally peer-reviewed.
